# S,S-Tetrazine-Based Hydrogels with Visible Light Cleavable Properties for On-Demand Anticancer Drug Delivery

**DOI:** 10.34133/2020/6563091

**Published:** 2020-09-04

**Authors:** Changping Wang, Chongyi Liu, Qiyao Wei, Lei Yang, Peng Yang, Yiwen Li, Yiyun Cheng

**Affiliations:** ^1^South China Advanced Institute for Soft Matter Science and Technology, South China University of Technology, Guangzhou 510640, China; ^2^Shanghai Key Laboratory of Regulatory Biology, School of Life Sciences, East China Normal University, Shanghai 200241, China; ^3^College of Polymer Science and Engineering, State Key Laboratory of Polymer Materials Engineering, Sichuan University, Chengdu 610065, China

## Abstract

Photocleavable hydrogels are of great importance in the field of controlled drug delivery, stem cell fate regulation, surface patterning, and intelligent devices. However, the development of novel photocleavable gel systems by visible light is usually met with challenges such as the lack of efficient and tunable photocleavable groups and reactions. Herein, we reported the facile fabrication of a new type of photocleavable hydrogels by the direct gelation of 4-arm thiol-terminated polyethylene glycol with 3,6-dichloro-1,2,4,5-tetrazine *via* the formation of S,S-tetrazine linkages. The prepared hydrogels underwent efficient degradation upon irradiation by ultraviolet or green light, and the degradation kinetics could be significantly promoted by hydrogen peroxide. Correspondingly, the hydrogels loaded with calcium peroxide microparticles or glucose oxidase/catalase enzymes enabled the precise and efficient *in vivo* photocontrol of gel degradation and drug release for cancer treatment. This work offers a promising and facile strategy towards the fabrication of visible light cleavable hydrogels with tunable and on-demand drug release properties.

## 1. Introduction

Smart hydrogels have attracted growing interest from the science community owing to their excellent biocompatibility with living systems and various promising applications ranging from on-demand drug delivery, cell fate regulation, self-healing, and shape memory materials to soft actuators [1–8]. For light-responsive hydrogels, the physicochemical, mechanical, and biological properties can be precisely controlled and rigorously tailored by light, a kind of stimulus with several unique advantages over others including being contact free, remote spatiotemporal regulation, safety, and low cost [9–13]. Although many of the efforts devoted to the design and fabrication of light-responsive hydrogels have been successful in proof-of-concept studies, their further usage in clinical applications was significantly hindered by profound challenges. One of the major concerns was related to the use of high-energy light, i.e., ultraviolet (UV), for most of the photochemical reactions or photoactivation processes. UV light usually showed poor tissue penetration ability, which failed to achieve photoactivated imaging and therapy in deep tissues. In addition, excessive UV light exposure could induce phototoxicity and other harmful side effects, resulting in damage to normal cells and tissues. One promising solution to overcome this issue typically focused on the fabrication of near-infrared (NIR) light-responsive systems, which could either undergo two-photon photolysis process [14, 15] or incorporate upconversion nanomaterials into the gel systems [16–18]. Although these gel systems show promising advantages, the total conversion efficiency of hydrogels by two-photon photolysis is limited by the narrow absorption cross-sections [9, 15]. In addition, these gel systems usually involve a sophisticated experimental set-up and redundant synthetic and purification work.

Visible light with specific wavelength regions such as green light (GL, 520-560 nm) has also been regarded as an alternative type of excitation light source due to its relatively deep tissue penetrability and minimally invasive feature [19–23]. The exploration of GL-responsive materials is currently in progress, and the discovery and screening of suitable photocleavable groups could play a critical role in the successful design of GL-cleavable hydrogels. The relevant photochemical reactions that can be triggered and activated by GL with rapid kinetics and high efficiency are of particular interest. Tetrazine-based chromophores have been well recognized as an important class of photochemical triggers used in biological systems since their photoproducts would not interfere with many biomacromolecules and cells [24–27]. In particular, recent advances in photochemistry and photobiology revealed that the S,S-tetrazine chromophore could be quickly photolyzed by UV and GL in a few ps, which has been successfully applied for the rapid tracking of the conformational dynamics of peptides, proteins, and synthetic macrocycles [28–34]. Although no responsive polymeric systems involving the S,S-tetrazine unit have been reported yet so far, we believe that this novel phototrigger should be a promising candidate to construct the desirable GL-cleavable hydrogels as proposed above.

In this work, we reported our first effort towards this goal by causing a 4-arm thiol-terminated polyethylene glycol (PEG) (4-arm-PEG-SH) to react with 3,6-dichloro-1,2,4,5-tetrazine (DT) to yield S,S-tetrazine chromophore crosslinked hydrogels. Those gels exhibited efficient degradation upon UV or GL irradiation and thereby released encapsulated cargoes. The light-triggered degradation and drug release behaviors could be significantly accelerated in the presence of hyperoxides. All the features of this hydrogel might allow the tunable and on-demand drug release *in vivo* with high spatial and temporal resolutions.

## 2. Results

**Figure 1:**
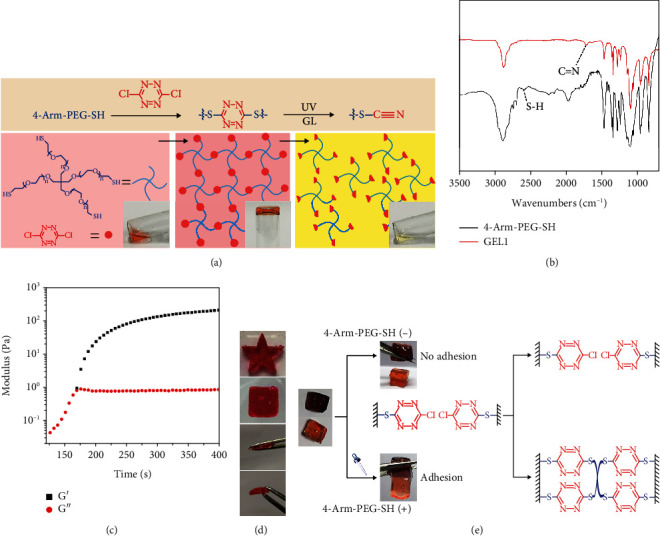
(a) Photographs, schematic illustration, and related mechanism of the hydrogel formation and degradation after light exposure. (b) FTIR spectra of GEL 1 and 4-arm-PEG-SH. (c) Dynamic rheology measurement of GEL 1. (d) Shaping and thixotropic test of GEL 1. (e) The adhesion testing and related mechanism of GEL 1 without/with the addition of 4-arm-PEG-SH.

Based on our material design, the hydrogel fabrication only requires the simple mixing of 4-arm-PEG-SH (20 mg/mL) with DT (1.25 mg/mL) in a PBS buffer. A red-colored hydrogel (GEL 1) was obtained within minutes *via* the formation of an S,S-tetrazine chromophore crosslinking network (Figure 1(a)). Note that the gel could be degraded under irradiation by UV or GL, which was attributed to the photocleavage of S,S-tetrazine chromophores. The Fourier transform infrared (FT-IR) spectrum showed the disappearance of the S-H stretching vibration band at 2588 cm^−1^ and the appearance of C=N stretching vibration at 1640 cm^−1^ (Figure 1(b)), confirming the successful crosslinking reactions during the gelation. Rheology study also exhibited a solution-to-gel transition after mixing the two species together (Figure 1(c)). The scanning electron microscopy (SEM) image in Figure S1 further suggested the fluffy microstructure of the formed GEL 1. In addition, the gel could be easily fabricated into several kinds of macroscopic shapes and showed excellent thixotropic properties (Figure 1(d)). Furthermore, two pieces of gels could be fused together by the addition of free 4-arm-PEG-SH, which was due to the interfacial formation of new S,S-tetrazine crosslinking networks between the gels (Figure 1(e)).

**Figure 2:**
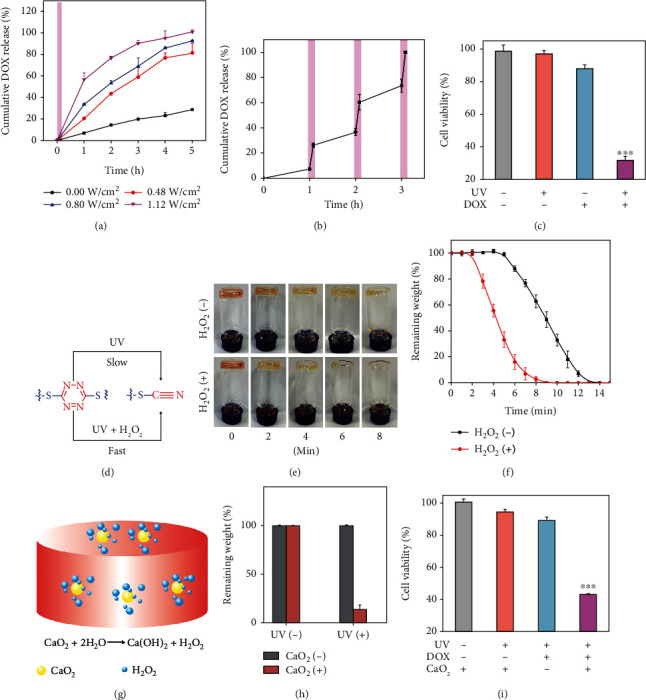
(a) Drug release profiles from GEL 1 loaded with DOX under UV irradiation at different power densities for 10 min. The purple band represents the UV irradiation process. (b) Drug release from GEL 1 loaded with DOX under periodic UV irradiation (5 min). The purple bands represent the 3 times UV irradiation process. (c) Viability of MDA-MB-231 cells treated with different hydrogels with/without UV irradiation. (d) Accelerated gel degradation by UV exposure in the presence of H_2_O_2_. (e) Photographs of GEL 1 under UV irradiation for 8 min in the presence or absence of H_2_O_2_. (f) Weight loss profiles of GEL 1 under UV irradiation in the presence or absence of H_2_O_2_. (g) Schematic illustration of CaO_2_-loaded hydrogel with sustained production of H_2_O_2_. (h) Weight loss of hydrogels under UV irradiation for 5 min in the presence or absence of CaO_2_. (i) Viability of MDA-MB-231 cells treated with different hydrogels under UV irradiation for 5 min.

To investigate the photocontrolled drug release behavior of the hydrogel, a typical anticancer drug doxorubicin (DOX) (0.125 mg/mL) was used as the model drug to be encapsulated in GEL 1 during gel formation in the PBS buffer. The drug release rate was increased in proportion to the power density of the UV light (Figure 2(a)) and could be tailored in an on-demand release manner by light irradiation (Figure 2(b)). We then tested the anticancer ability of released drugs from the gel on MDA-MB-231 cells. The released DOX from GEL 1 were collected after light exposure (1.12 W/cm^2^, 15 min) for toxicity assay. UV light irradiation significantly increased the amount of released DOX from the gel and thereby caused significant toxicity on the treated MDA-MB-231 cells (Figures 2(c) and S2). In comparison, the nonirradiated gel loaded with DOX showed minimal toxicity on the cells under the same condition. These results suggested that the S,S-tetrazine-based hydrogel could be used for light-controlled drug release. More interestingly, previous work reported that the photolysis of S,S-tetrazine can be accelerated by oxygen and hyperoxide [29]. We therefore investigated the effect of hyperoxide such as hydrogen peroxide (H_2_O_2_) on the photodegradation kinetics of the hydrogels (Figure 2(d)). Digital photographs of GEL 1 by directly treating with and without H_2_O_2_ upon UV irradiation (1.12 W/cm^2^) were recorded in Figure 2(e). GEL 1 was completely degraded after UV irradiation for 6 min in the presence of H_2_O_2_, while lots of gels remained in the bottle even after 8 min of irradiation in the absence of H_2_O_2_. Correspondingly, the quantitative weight loss kinetic curves of GEL 1 with or without H_2_O_2_ were also monitored (Figure 2(f)), and the results further confirmed the accelerated gel photolysis by H_2_O_2_. Since H_2_O_2_ could be rapidly leaked out of the hydrogel, we further optimized the gel system by incorporating with CaO_2_ microparticles, which could consistently generate H_2_O_2_ by hydrolysis (Figure 2(g)). CaO_2_ (0.42 mg/mL) was loaded into the gel during gelation, and the obtained hydrogel was termed GEL 2. Note that the presence of CaO_2_ particles did not affect the gel formation process (Figure S3a), and these particles were randomly distributed in the gel network (Figure S3b). After UV irradiation (1.12 W/cm^2^) for 5 min, about 80% of GEL 2 were degraded, while most of GEL 1 without CaO_2_ particles remained in the gel state (Figure 2(h)). Similarly, UV light-triggered release of DOX from GEL 2 (1.12 W/cm^2^, 5 min) induced significant toxicity on MDA-MB-231 cells (Figure 2(i)). Notably, the photocleavage of S,S-tetrazine crosslinking units in the hydrogel only yielded inert PEG analogues and nitrogen gas, and the products as well as loaded CaO_2_ caused minimal toxicity to biological tissues. All the components (4-arm-PEG-SH, DT, and CaO_2_) as well as GEL 2 showed extremely low toxicity on NIH3T3 cells (Figure S4).

**Figure 3:**
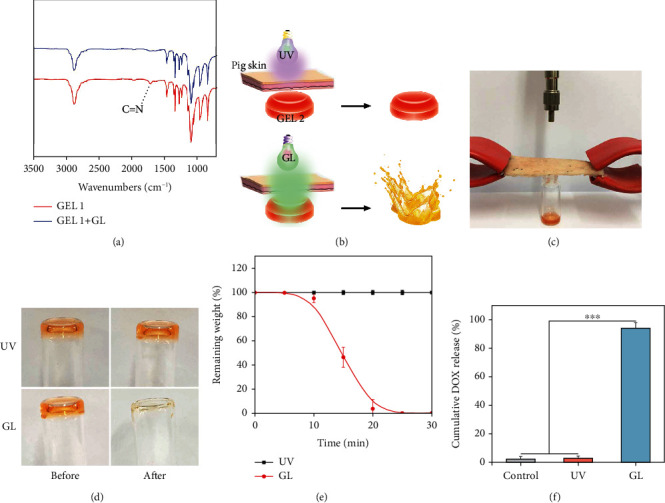
(a) FTIR spectra of GEL 1 before and after GL irradiation. Schematic illustration (b) and experiment model (c) of light-triggered degradation of hydrogels covered by a pig skin. (d) Photographs of pig skin-covered GEL 2 before and after light irradiation (1.12 W/cm^2^, 20 min). (e) Weight loss profiles of GEL 2 after irradiation by UV or GL for different times. (f) The cumulative drug release from GEL 2 after light irradiation for 20 min.

Although S,S-tetrazine crosslinked hydrogels showed photodegradable properties under UV exposure, *in vivo* applications of those hydrogels remain a challenging problem due to the weak penetration and phototoxicity of UV light. It was observed that GL could not induce any phototoxicity to NIH3T3 cells even after 10 min of irradiation, as evidenced by the cell viability result shown in Figure S5. Based on this result, we then investigated the possibility of using GL (532 nm, 1.12 W/cm^2^) to remotely control the degradation of GEL 2. The characteristic peak at 1640 cm^−1^ attributed to the C=N stretching vibration in the FTIR spectrum was significantly decreased after irradiation, which confirmed the cleavage of S,S-tetrazine chromophores in the gel by GL (Figure 3(a)). GEL 2 could also be degraded under GL irradiation, and the drug release rate was increased in proportion to the power density of the GL (Figure S6). A tiny piece of pig skin (40 mm × 20 mm × 2 mm) was covered on GEL 2 to investigate the photodegradation behaviors under exposure to UV and GL at the same power density (1.12 W/cm^2^, Figures 3(b) and 3(c)). Note that the pig skin purchased from the food market was widely employed in many established optical studies due to its similar anatomical and optical properties with normal human skin [35, 36]. As shown in Figure 3(d), only GL was able to trigger the degradation of GEL 2 covered with a pig skin after irradiation. This conclusion was also supported by the corresponding weight loss of GEL 2 during light exposure (1.12 W/cm^2^, Figure 3(e)). Subsequently, GL was also observed to induce efficient DOX release from the hydrogel covered by a pig skin, but UV light at an equal power density failed to trigger efficient drug release (Figure 3(f)). These results encouraged us to test the possibility of this gel system for *in vivo* drug delivery applications.

**Figure 4:**
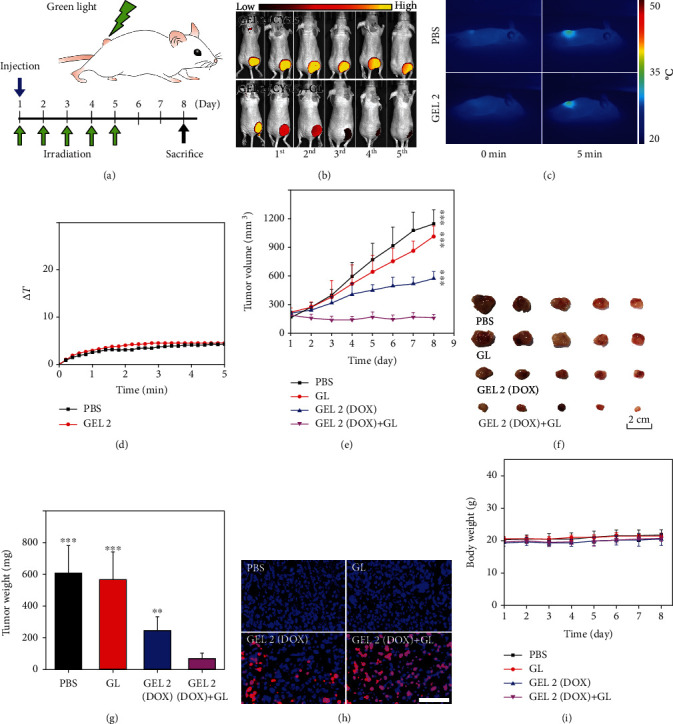
(a) Schematic illustration of the therapeutic experiment. (b) Fluorescence imaging of mice injected with CY5.5-loaded GEL 2 with or without GL irradiation. Thermographs (c) and temperature evolution (d) of the mice injected with GEL 2 after GL irradiation. The tumor volume changes (e), excised tumor images (f), and tumor weight analysis (g) of mice in different groups. (h) Apoptosis (red) in tumors of the mice analyzed by TUNEL assay. The cell nuclei were labeled with Hoechst 33342 (blue). Scale bars: 100 *μ*m. (i) Body weight change curves of the mice after different treatments.

We then tested the *in vivo* light-responsive behaviors of the developed hydrogels (Figure 4(a)). The *in vivo* photodegradation behavior of GEL 2 was firstly confirmed by the use of NIR dye cyanine 5.5 (CY5.5) as the model cargo. GL irradiation could efficiently induce the release of CY5.5 from the injected gel site as observed by an *in vivo* imaging system (IVIS, Figure 4(b)). We further tested the therapeutic efficiency of DOX-loaded GEL 2 on a subcutaneous tumor model. Twenty nude mice bearing subcutaneous MDA-MB -231 tumors were randomly divided into two groups. The mice in the two groups were injected with PBS and DOX-loaded GEL 2, respectively. Five of the mice in each group were irradiated with GL (1.12 W/cm^2^, 5 min) as described in the therapeutic schedule (Figure 4(a)), and the remaining mice were not further treated. During the irradiation period, no significant hyperthermia effect was observed based on the thermographs (Figures 4(c) and 4(d)). After treatment, the mice in the GEL group combined with GL exposure showed significantly reduced tumor growth (Figures 4(e)–4(g)), while those in the nonirradiated gel group only displayed a moderate therapeutic efficiency. Meanwhile, the GL-irradiated PBS group did not exhibit efficient tumor inhibition. These results were in good agreement with the apoptosis analysis of tumors by terminal deoxynucleotidyl transferase dUTP nick end labeling (TUNEL) assay (Figure 4(h)). Additionally, there were no obvious body weight changes for the mice during the treatment, suggesting minimal systemic toxicity of the gel and GL treatment (Figure 4(i)).

**Figure 5:**
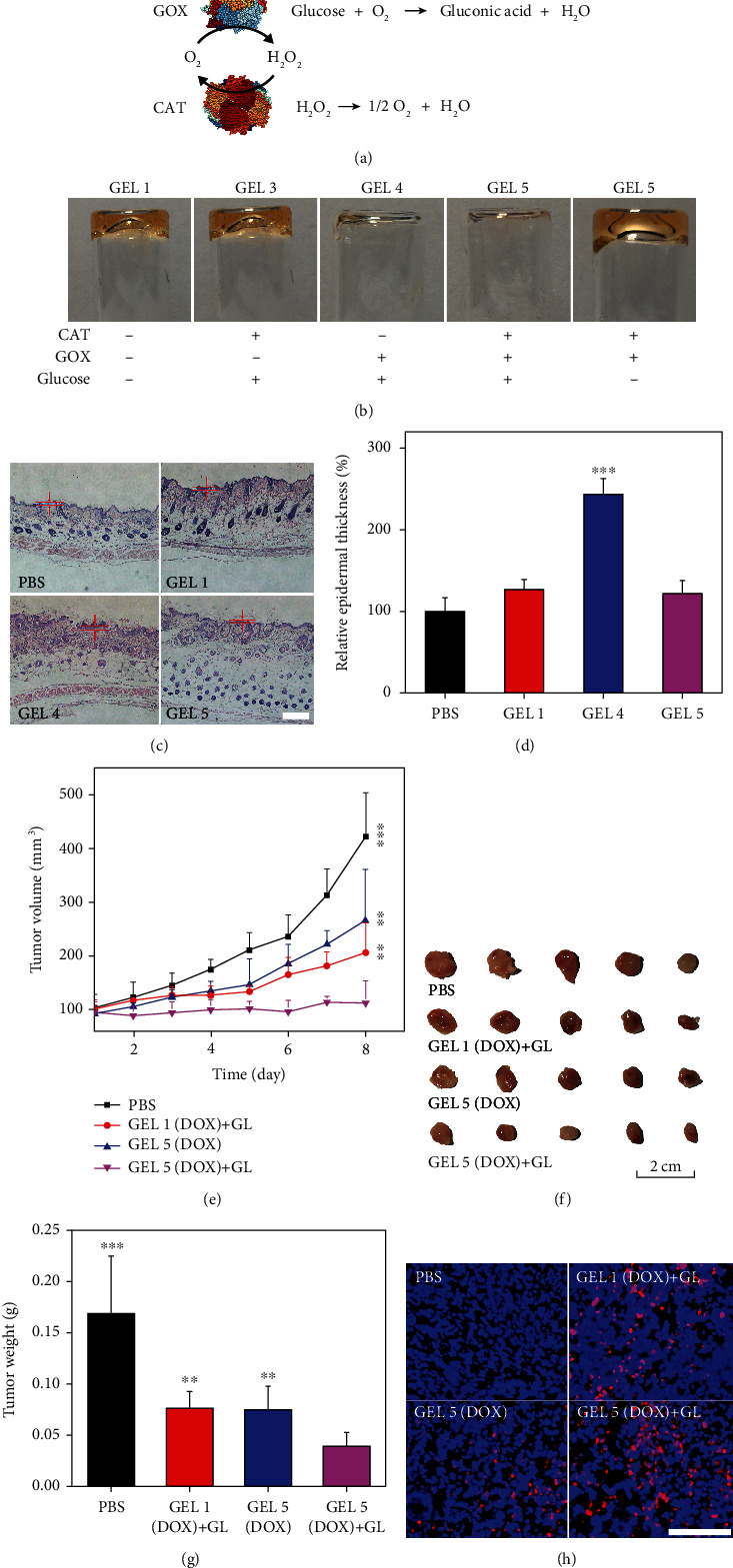
(a) Schematic representation of the GOX/CAT enzyme system. (b) Digital photographs of GEL 1, GEL 3, GEL 4, and GEL 5 with different components after GL exposure. (c) H&E staining of skin tissues treated with different gels. Scale bars: 100 *μ*m. (d) Relative epidermal thickness of the skin treated with different gels. (e) The size of the tumor in different groups during the treatment. (f) Photographs of the excised tumors. (g) Average weight of the excised tumors. (h) Apoptosis (red) in tumors of mice after treatment. The cell nuclei were labeled with Hoechst 33342 (blue). Scale bars: 100 *μ*m.

It was reported that cascade enzymatic reactions by glucose oxidase (GOX) and catalase (CAT) can also produce H_2_O_2_ and O_2_ under physiological conditions (Figure 5(a)) [37, 38]. To further expand the scope of the hydrogels for *in vivo* applications, we proposed the use of a GOX/CAT enzyme system to accelerate the photodegradation of the S,S-tetrazine-based hydrogel in the presence of glucose. GOX could oxidize the glucose to produce H_2_O_2_, and CAT scavenged the excessive H_2_O_2_ into H_2_O and O_2_, thus minimizing the risk of adverse effects caused by H_2_O_2_. GEL 1 loaded with CAT (0.5 mg/mL), GOX (0.5 mg/mL), and GOX/CAT (0.5 mg/mL GOX and 0.5 mg/mL CAT) were termed GEL 3, GEL 4, and GEL 5 in this study, respectively. Both GEL 4 and GEL 5 showed efficient gel degradation after GL irradiation (1.12 W/cm^2^, 5 min); in comparison, GEL 1 or GEL 3 failed to exhibit photodegradation behaviors under the same condition (Figure 5(b)). The GOX substrate glucose also played a critical role in gel photodegradation. The generated H_2_O_2_ from different gels was measured by a Catalase Assay Kit, and it could be found that the generation of H_2_O_2_ from GEL 5 was rigorously controlled in a steady state (Figure S7). In contrast, the H_2_O_2_ production from GEL 4 was much faster and ~5 mM H_2_O_2_ can be accumulated after a 3 h reaction. We then examined the toxicity of GEL 4 and GEL 5 on skin tissues after subcutaneous injection. PBS and GEL 1 were tested as controls (Figures 5(c) and 5(d)). GEL 4 treatment caused significant increase in the thickness of the stratum corneum due to the excessive H_2_O_2_ produced by GOX; in comparison, GEL 1 and GEL 5 did not cause obvious change in the thickness, suggesting the essential role of CAT in reducing the side effects of GOX [39, 40]. Finally, we examined the therapeutic efficacy of GEL 5 loaded with DOX *in vivo*. The therapeutic schedule was the same as what is described in Figure 4(a). DOX-loaded GEL 5 combined with GL treatment showed the most efficient anticancer ability with minimal adverse effects (Figures 5(e)–5(g) and Figure S8). This conclusion was also supported by the apoptosis analysis by TUNEL assay (Figure 5(h)).

## 3. Discussion

In summary, we designed and optimized a new type of photocleavable hydrogels based on S,S-tetrazine linkage for on-demand drug delivery. The gel fabrication process is quite simple, and the S,S-tetrazine linkages in the hydrogel could undergo efficient photocleavage upon GL and UV irradiation. The prepared hydrogels also demonstrated efficient *in vivo* photocontrol of gel degradation and drug release with hyperoxide-accelerated behaviors, successfully realizing promising antitumor therapeutic performances. We believe that this study would stimulate the further exploration of more types of visible light-responsive implant soft devices with tunable functionalities for precise *in vivo* therapies.

## 4. Materials and Methods

### 4.1. Materials and Chemicals

4-Arm-PEG-SH (20 kDa) was purchased from JenKem Technology (Beijing, China). DT (96%) was purchased from Sigma-Aldrich (St. Louis, MO). DOX, GOX, CAT, and H_2_O_2_ (AR, 30 wt.%) were obtained from ALADDIN Reagent (Shanghai, China). CaO_2_ was purchased from Alfa Aesar (Ward Hill, MA). 3-(4,5-Dimethylthiazol-2-yl)-2,5-diphenyltetrazolium bromide (MTT, 98%) and acridine orange (AO) were bought from Sangon Biotech (Shanghai, China). Ethidium bromide (EB) was purchased from TIANGEN Biotech (Beijing, China). CY5.5 was obtained from Lumiprobe Corporation (Florida, USA). A TUNEL Apoptosis Assay Kit and Proteinase K were from Roche Diagnostics (Mannheim, Germany).

### 4.2. Hydrogel Formation

For GEL 1, 4-arm-PEG-SH was suspended in PBS (pH 7.4, 0.01 M) buffer at a concentration of 80 mg/mL, and DT was dissolved in PBS at a concentration of 2 mg/mL. Then, 4-arm-PEG-SH, DT, and PBS solutions were mixed at the volume ratio of 2 : 5 : 1. The solutions were homogeneously mixed to prepare GEL 1. For GELs 2-5, the preparation protocols were similar to GEL 1 except for the addition of more components during gel formation. The resulting gels need to be washed several times to remove all the small molecular residuals. GEL 1 with different shapes was formed by in situ gelation in silicone molds with different shapes. The detailed formulas of GELs 1-5 are listed in Table S1.

### 4.3. Characterization

The SEM image was captured by using a Hitachi microscope (S-4800, Hitachi, Japan) operated at 10 kv. High-performance liquid chromatography (HPLC) was conducted on an Agilent 1200 equipped with a C18 column (4.6 mm diameter, 150 mm length, 5 mm particle size, ZORBAX Eclipse XDB, Agilent, USA). The FT-IR spectra were measured by a Fourier transform infrared spectrometer (Thermo Scientific, Nicolet iS50, USA). The dynamic rheology measurement was carried out using a hybrid rheometer (Discovery Hybrid Rheometer-3, TA Instruments, USA). The thermographs and temperatures were recorded using an infrared camera (Magnity Electronics, China).

### 4.4. UV Light-Triggered Hydrogel Degradation

GEL 1 formed in glass vials was irradiated by a UV laser at a power density of 1.12 W/cm^2^ for 15 min, and the vials were sloped to a certain angle to observe the gel state. To determine the degradation percentage of hydrogels, the as-generated solutions in the vials after UV irradiation were removed, and the remaining hydrogels were then weighed. The degradation kinetics of GEL 1 in the presence of 10 mM H_2_O_2_ was investigated by the same procedure. Digital photographs and weight loss of the gels were recorded every minute during UV irradiation. GEL 1 in the absence of H_2_O_2_ was tested as a control.

### 4.5. In Vitro Drug Release Kinetics Assay

DOX was used as a model drug in the light-triggered drug release assay. GEL 1 (0.12 mL) loaded with DOX (0.125 mg/mL) was irradiated by UV light at different power densities (0~1.12 W/cm^2^) for 10 min, and then 2 mL fresh PBS buffer was added to dissolve the released ingredients. A 10 *μ*L solution was collected at the different time points of 0, 1, 2, 3, 4, and 5 h, respectively. For each collection of the above solution, equal volume of free PBS was fed back. The DOX concentration in the collected samples was measured by HPLC. The used gradient was the mixture of acetonitrile and 0.2% trifluoroacetic acid solution at a volume ratio of 35 : 65, and the flow rate was 1.0 mL/min. The wavelength of the detector was set as 490 nm. The drug release studies under periodic UV irradiation were conducted by a similar procedure. DOX-loaded GEL 1 was irradiated by UV light at a power density of 1.12 W/cm^2^ for 5 min at the schedule of 1, 2, and 3 h, respectively. Then, the released drugs in the collected samples were analyzed by HPLC.

### 4.6. Cell Culture

MDA-MB-231 cells (a human breast cancer cell line, ATCC) were cultured at 37°C in MEM medium (GIBCO) supplemented with 10% fetal bovine serum (FBS, Gemini), 100 *μ*g/mL streptomycin, and 100 *μ*g/mL penicillin. NIH3T3 cells (a mouse embryo fibroblast cell line, ATCC) were cultured in DMEM (GIBCO) containing 10% FBS, 100 *μ*g/mL streptomycin, and 100 *μ*g/mL penicillin.

### 4.7. Cell Viability Assay

The cytotoxicity of the gel components including 4-arm-PEG-SH, DT, and CaO_2_ as well as the prepared hydrogels (GEL 1 and GEL 2) was analyzed on NIH3T3 cells. The cells were incubated in 96-well plates overnight at 37°C. Different concentrations of 4-arm-PEG-SH (0.05, 0.1, 0.5, 1, and 2 mg/mL), DT (2, 4, 6, 8, and 10 *μ*g/mL), or CaO_2_ (1, 5, 10, 20, and 30 *μ*g/mL) were added into the culture media and then incubated for 48 h at 37°C. After that, a standard 3-(4,5-dimethylthiazol-2-yl)-2,5-diphenyltetrazolium bromide (MTT) assay was carried out to determine the cell viability. To determine the cytotoxicity of GEL 1 and GEL 2 upon UV irradiation, 0.12 mL GEL 1 and GEL 2 was irradiated by UV light at a power density of 1.12 W/cm^2^ for 15 min, and then 200 *μ*L PBS was added to dissolve the released ingredients. 1 *μ*L of the solution was collected and incubated with NIH3T3 cells. After 48 h, the viability of treated cells was determined as described above. GEL 1 and GEL 2 without UV irradiation were tested as controls.

The cytotoxicity of released drugs from the hydrogel was also evaluated by MTT assay. Generally, MDA-MB-231 cells were incubated in 96-well plates overnight at 37°C. 0.12 mL GEL 1 loaded with DOX (0.125 mg/mL) was irradiated by UV light at a power density of 1.12 W/cm^2^ for 15 min, and then 200 *μ*L PBS was added to dissolve the released DOX. 1 *μ*L of the solution was collected and added into the wells. After incubation for 48 h, the standard MTT assay was carried out to determine the cell viability. Nonirradiated and irradiated GEL 1, as well as DOX-loaded GEL 1 without UV irradiation, were tested as controls. The cytotoxicity of the released drug on MDA-MB-231 cells was also evaluated by an AO/EB staining assay. The cells were treated with released DOX samples as described above, and the cells were stained with AO (5 *μ*g/mL) and EB (5 *μ*g/mL) for 10 min and imaged by a fluorescence microscope (Olympus, Japan).

### 4.8. Photodegradation of Hydrogels Covered by a Pig Skin

Pig skin purchased from a food market was employed as a model to mimic the human skin tissue. A piece of pig skin (40 mm × 20 mm × 2 mm) was covered on the surface of GEL 2. Then, the gel was irradiated by UV light (1.12 W/cm^2^) or GL (1.12 W/cm^2^) for 30 min. Digital photographs and weight loss of the irradiated gels were recorded every 5 min.

### 4.9. In Vivo Drug Release Experiments

Five-week-old male BALB/c nude mice with an average weight of 20 g were purchased from the animal center of East China Normal University (ECNU). All the animal experiments were approved by the animal experimentation ethics committee of ECNU. The MDA-MB-231 tumor xenograft model was established by subcutaneous injection of MDA-MB-231 cells (~10^6^ cells) suspended in 100 *μ*L PBS into the right back of mice.

Two mice were chosen for the *in vivo* drug release experiment. 30 *μ*L GEL 2 loaded with 8.3 *μ*g/mL CY5.5 was injected into the right back of mice. IVIS images of the mice were recorded 30 min after the injection. Then, one of the mice was treated with GL irradiation at a power density of 1.12 W/cm^2^ for 5 min every day, and the treatment was repeated for five times. The other animal was not treated with light irradiation during the period. IVIS images of the mice were captured every day after GL irradiation to determine the release of CY5.5 from the irradiated GEL 2.

Then, the mice with an average tumor volume of 200 mm^3^ were randomly divided into four groups with five mice in each group. 30 *μ*L GEL 2 loaded with 1.25 mg/mL DOX was injected into the tumors of two groups of mice. One group of mice was treated with GL irradiation on the tumor site at a power density of 1.12 W/cm^2^ for 5 min every day, and five irradiations were treated. The other group of mice did not receive any light irradiation. The other two groups of mice were intratumorally injected with 30 *μ*L PBS, and the mice were treated without or with GL irradiation as described above. The tumor volumes and body weights of mice were recorded every day during the treatment. At the 8^th^ day, the tumor bearing mice were sacrificed, and tumors were excised and weighted.

The *in vivo* enzyme-enhanced GEL degradation assay was conducted under the same condition. The mice with an average tumor volume of 100 mm^3^ were randomly divided into four groups with five mice in each group. 30 *μ*L GEL 5 loaded with 1.25 mg/mL DOX was intratumorally injected into the tumor site, and the mice were irradiated by GL irradiation at a power density of 1.12 W/cm^2^ for 5 min every day, and in total, five irradiations were treated. The other three groups of mice were treated with PBS, DOX-loaded GEL 1 with GL irradiation, and DOX-loaded GEL 5 without GL irradiation. The tumor volumes and body weights of mice were recorded every day. At the 8^th^ day, the tumor bearing mice were sacrificed and tumors were excised and weighted.

### 4.10. TUNEL Assay

The tumor bearing mice were sacrificed after treatment, and the tumor tissues were harvested and fixed in 4% formalin solution at 4°C overnight and sectioned into 5 *μ*m slices. The tumor sections were then incubated with proteinase K, the TUNEL reaction mixture, and Hoechst 33342 according to the protocols of the in situ apoptosis detection kit (Roche, Mannheim Germany). Apoptotic cells in the sections were imaged with a fluorescence microscope.

### 4.11. Dermal Toxicity of the Hydrogels

30 *μ*L GEL 1, GEL 4, and GEL 5 was subcutaneously injected into the right back of mice. PBS was used as a control. Each group contains three mice. After the gels were injected for eight days, skin tissues at the injection area were collected for histological examination. The skin samples were fixed in 4% formalin solution at 4°C overnight and sectioned into 5 *μ*m slices. The sections were deparaffinized by xylene, followed by rehydration and then staining with hematoxylin and eosin.

## Supplementary Materials

Supplementary Materials Table S1: the detailed formula in GEL 1-5 (mg/mL). Figure S1: SEM image of GEL 1. Figure S2: cytotoxicity of the released drug determined by the AO/EB staining assay on MDA-MB-231 cells. Scale bars: 100 *μ*m. Figure S3: (a) GEL 2 formation revealed by the dynamic development of G′ and G^″^ moduli over time. (b) SEM image of GEL 2. Figure S4: cytotoxicity of 4-arm-PEG-SH (a), DT (b), CaO_2_ (c), and GEL 2 (d) on NIH3T3 cells. (e) The biocompatibility of the 4-arm-PEG-SH, DT, CaO_2_, and GEL 2 revealed by AO/EB staining assay on NIH3T3 cells. Scale bars: 400 *μ*m. Figure S5: cell viability of NIH3T3 cells after 10 min UV/GL irradiation at a power density of 0.48 W/cm^2^. Figure S6: (a) weight loss profiles of GEL 2 under GL irradiation at a power density of 1.12 W/cm^2^. (b) Drug release profiles from GEL 2 loaded with DOX under GL at different power densities for 5 min. Figure S7: glucose-dependent generation of H_2_O_2_. Figure S8: body weight change curves of the mice after different treatments.
