# Supplementary material for: S,S-Tetrazine-Based Hydrogels with Visible Light Cleavable Properties for On-Demand Anticancer Drug Delivery
Source: Research (Wash D C). 2020 Sep 4;2020:6563091. doi: 10.34133/2020/6563091 (PMC7510344; doi:10.34133/2020/6563091)
Supplement: Supplementary Materials — Table S1: the detailed formula in GEL 1-5 (mg/mL). Figure S1: SEM image of GEL 1. Figure S2: cytotoxicity of the released drug determined by the AO/EB staining assay on MDA-MB-231 cells. Scale bars: 100 μm. Figure S3: (a) GEL 2 formation revealed by the dynamic development of G′ and G″ moduli over time. (b) SEM image of GEL 2. Figure S4: cytotoxicity of 4-arm-PEG-SH (a), DT (b), CaO2 (c), and GEL 2 (d) on NIH3T3 cells. (e) The biocompatibility of the 4-arm-PEG-SH, DT, CaO2, and GEL 2 revealed by AO/EB staining assay on NIH3T3 cells. Scale bars: 400 μm. Figure S5: cell viability of NIH3T3 cells after 10 min UV/GL irradiation at a power density of 0.48 W/cm2. Figure S6: (a) weight loss profiles of GEL 2 under GL irradiation at a power density of 1.12 W/cm2. (b) Drug release profiles from GEL 2 loaded with DOX under GL at different power densities for 5 min. Figure S7: glucose-dependent generation of H2O2. Figure S8: body weight change curves of the mice after different treatments. [file 6563091.f1.docx]

Supporting information

S,S-tetrazine based hydrogels with visible light cleavable properties for on demand anticancer drug delivery

Changping Wang^1, ‡^, Chongyi Liu^2, ‡^, Qiyao Wei^2^, Lei Yang^3^, Peng Yang^3^, Yiwen Li^3,^* and Yiyun Cheng^1, 2,^*

^1^South China Advanced Institute for Soft Matter Science and Technology, South China University of Technology, Guangzhou 510640, P.R. China.

^2^Shanghai Key Laboratory of Regulatory Biology, School of Life Sciences, East China Normal University, Shanghai, 200241, P.R. China.

^3^College of Polymer Science and Engineering, State Key Laboratory of Polymer Materials Engineering, Sichuan University, Chengdu 610065, China.

E-mail: yycheng@mail.ustc.edu.cn (Y.C.), Tel: +86 021-54341001; E-mail: [ywli@scu.edu.cn](mailto:ywli@scu.edu.cn) (Y.L.), Tel: +86 028-85401066.

^‡^ These authors contributed equally to this work.

**Table S1.** The detailed formula in GEL 1-5 (mg/mL).


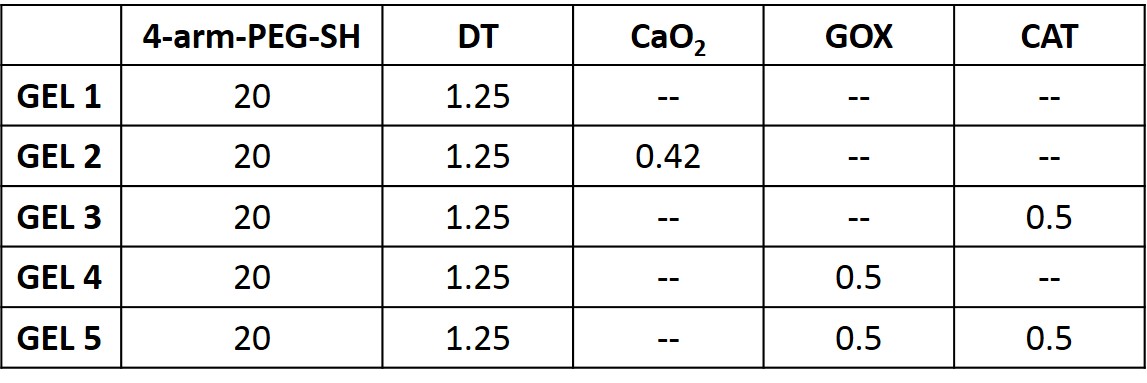


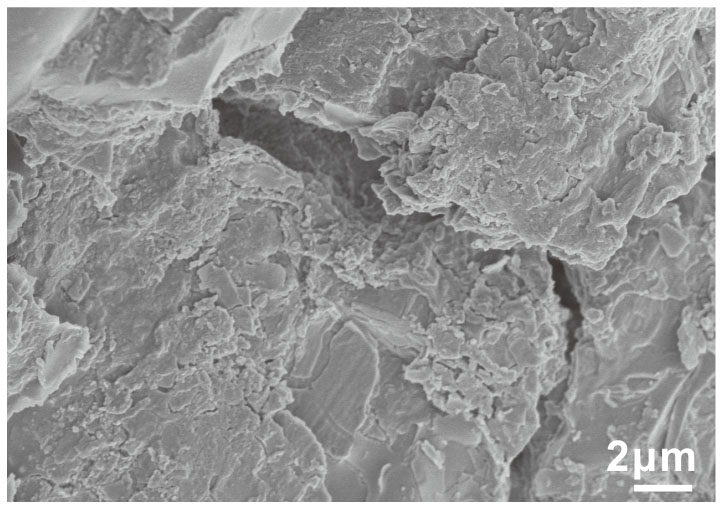


**Figure S1.** SEM image of GEL 1.


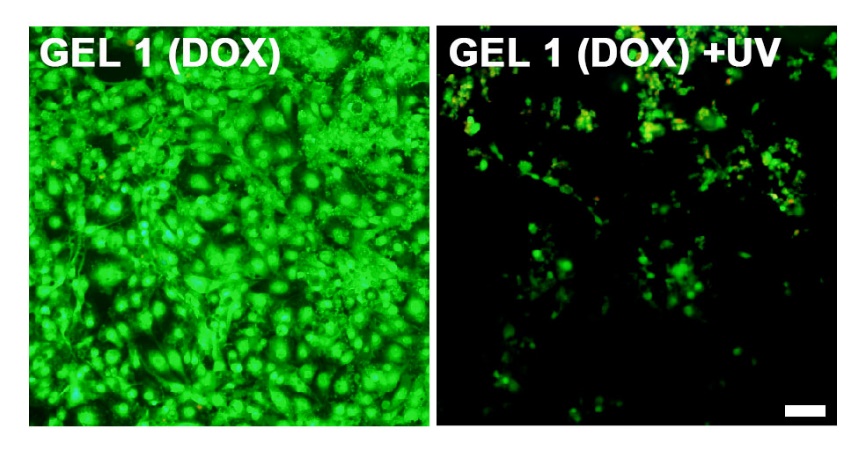


**Figure S2.** Cytotoxicity of the released drug determined by the AO/EB staining assay on MDA-MB-231 cells. Scale bars: 100 μm.


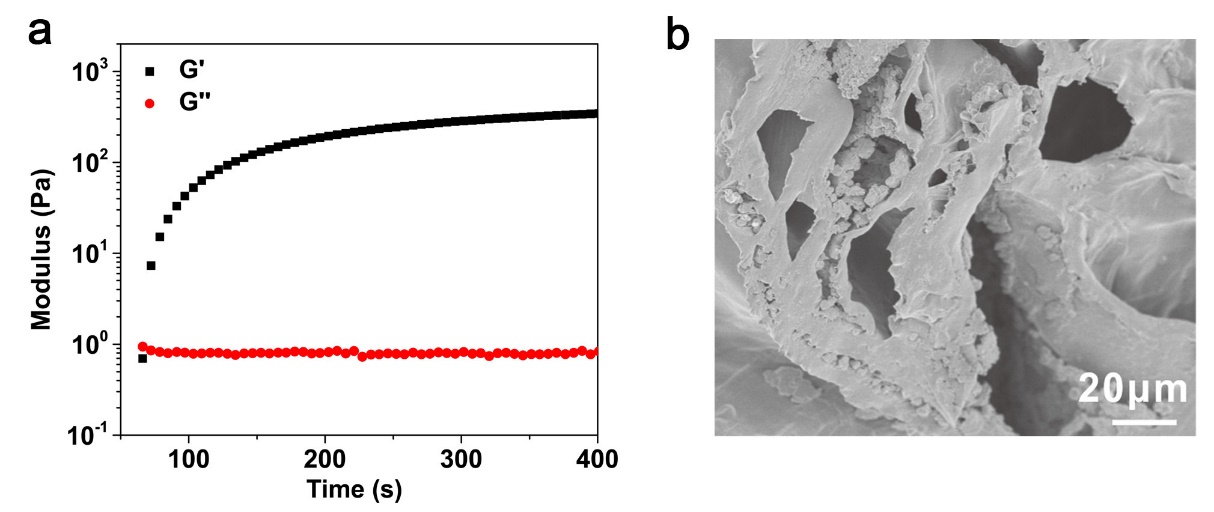


**Figure S3. (**a) GEL 2 formation revealed by the dynamic development of G' and G'' moduli over time. (b) SEM image of GEL 2.


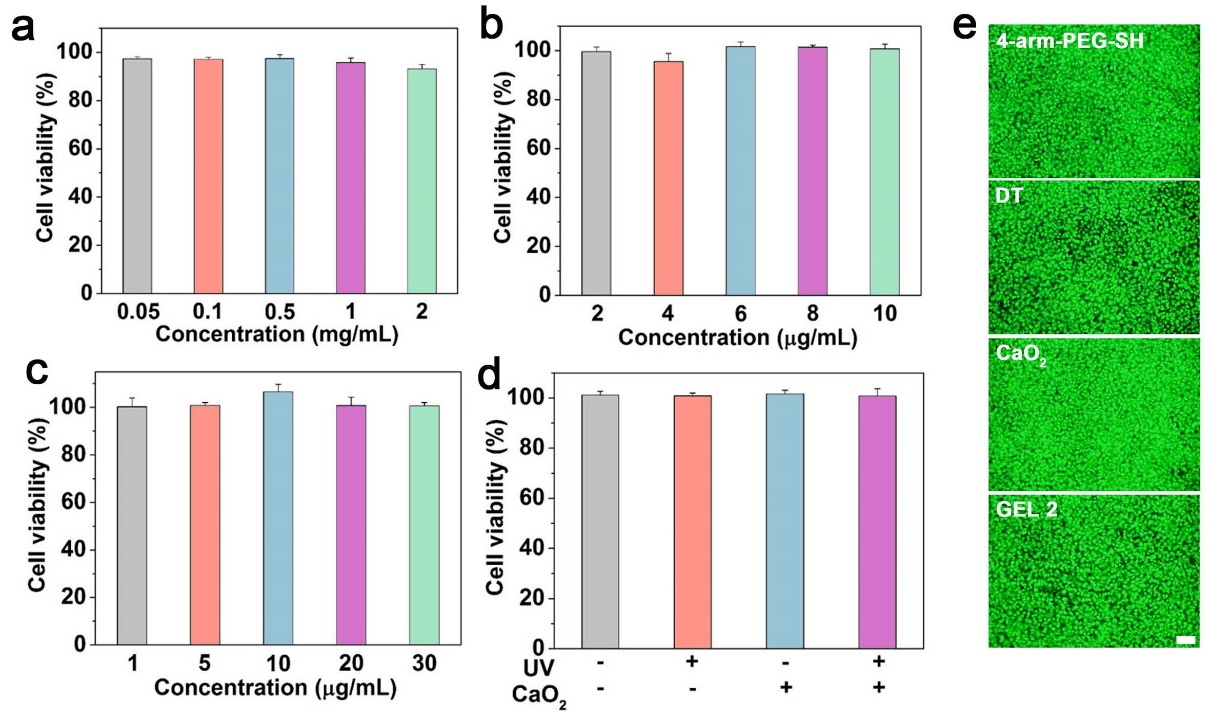


**Figure S4.** Cytotoxicity of 4-arm-PEG-SH (a), DT (b), CaO_2_ (c), and GEL 2 (d) on NIH3T3 cells. (e) The biocompatibility of the 4-arm-PEG-SH, DT, CaO_2_, and GEL 2 revealed by AO/EB staining assay on NIH 3T3 cells. Scale bars: 400 μm.


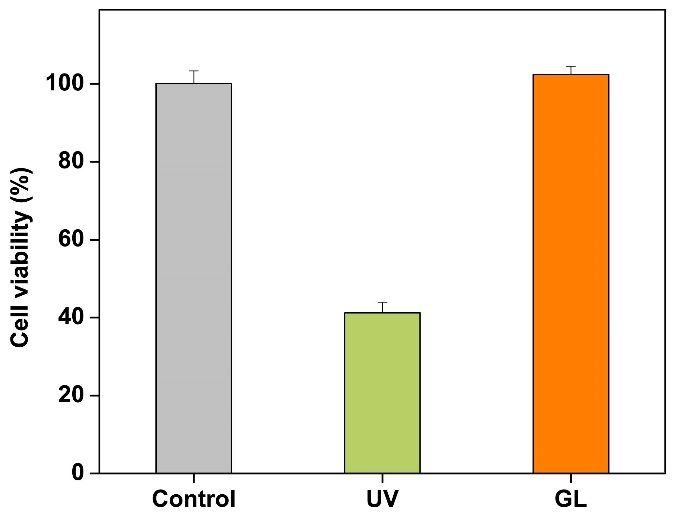


Figure S5. Cell viability of NIH 3T3 cells after 10 min UV/GL irradiation at a power density of 0.48 W/cm^2^.


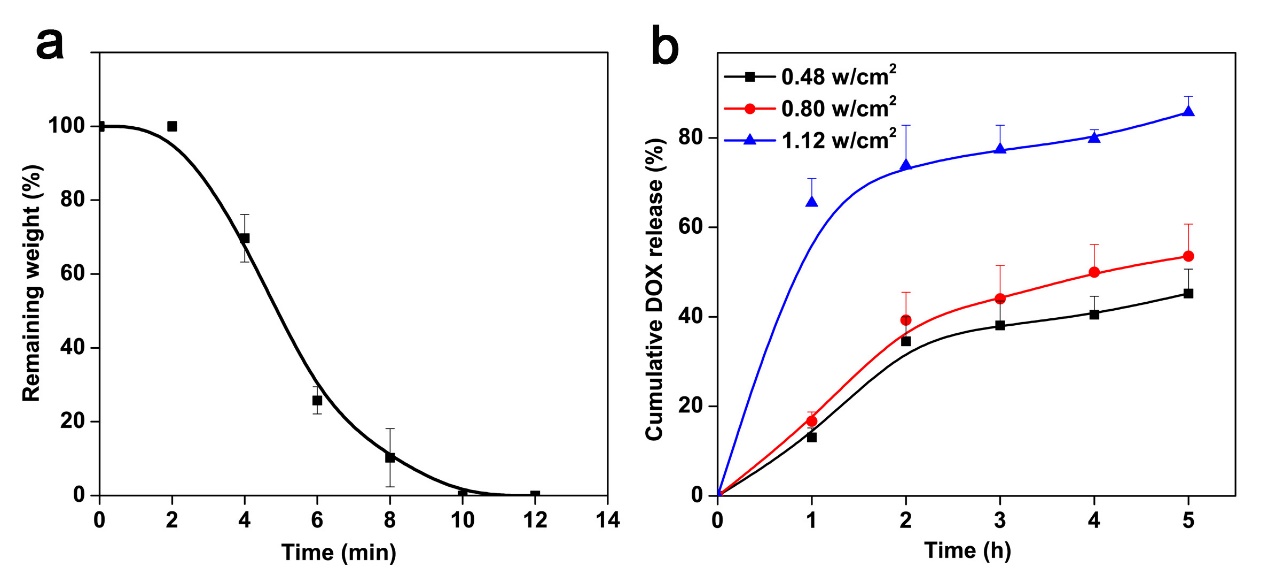


Figure S6. (a) Weight loss profile of GEL 2 under GL irradiation at a power density of 1.12 W/cm^2^. (b) Drug release profiles from GEL 2 loaded with DOX under GL at different power densities for 5 min.


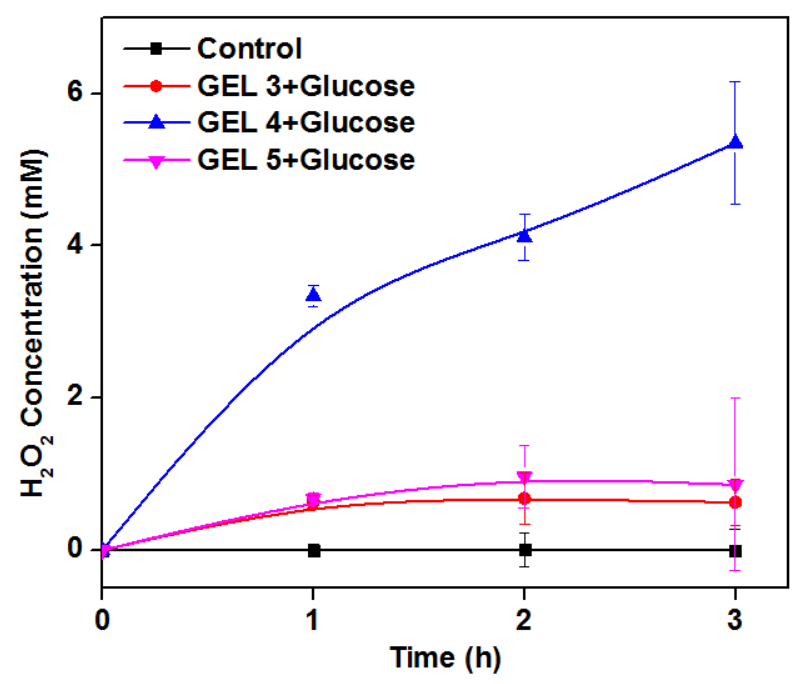


**Figure S7** Glucose-dependent generation of H_2_O_2_.


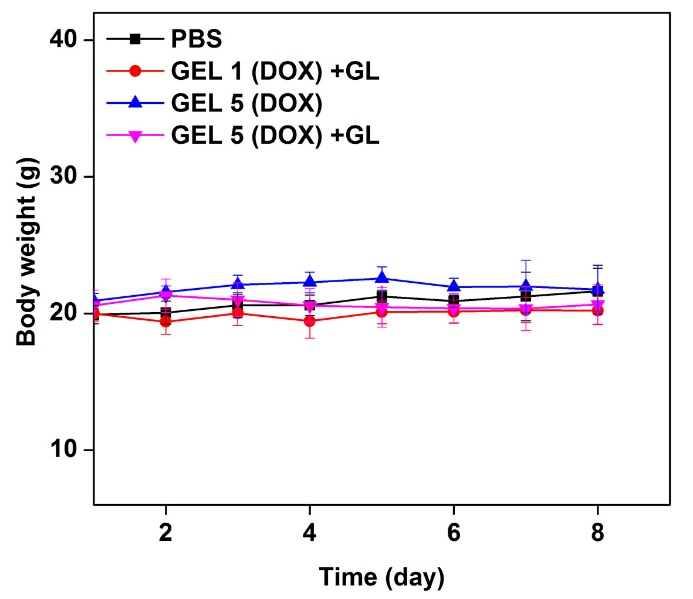


**Figure S8.** Body weight change curves of the mice after different treatments.
